# The Effect of Spatial and Temporal Resolution of Cine Phase Contrast MRI on Wall Shear Stress and Oscillatory Shear Index Assessment

**DOI:** 10.1371/journal.pone.0163316

**Published:** 2016-09-26

**Authors:** Merih Cibis, Wouter V. Potters, Frank J. Gijsen, Henk Marquering, Pim van Ooij, Ed vanBavel, Jolanda J. Wentzel, Aart J. Nederveen

**Affiliations:** 1 Biomedical Engineering Department, Erasmus MC, Rotterdam, The Netherlands; 2 Radiology Department, Academic Medical Center (AMC), Amsterdam, The Netherlands; 3 Biomedical Engineering and Physics Department, AMC, Amsterdam, The Netherlands; University of California San Diego, UNITED STATES

## Abstract

**Introduction:**

Wall shear stress (WSS) and oscillatory shear index (OSI) are associated with atherosclerotic disease. Both parameters are derived from blood velocities, which can be measured with phase-contrast MRI (PC-MRI). Limitations in spatiotemporal resolution of PC-MRI are known to affect these measurements. Our aim was to investigate the effect of spatiotemporal resolution using a carotid artery phantom.

**Methods:**

A carotid artery phantom was connected to a flow set-up supplying pulsatile flow. MRI measurement planes were placed at the common carotid artery (CCA) and internal carotid artery (ICA). Two-dimensional PC-MRI measurements were performed with thirty different spatiotemporal resolution settings. The MRI flow measurement was validated with ultrasound probe measurements. Mean flow, peak flow, flow waveform, WSS and OSI were compared for these spatiotemporal resolutions using regression analysis. The slopes of the regression lines were reported in %/mm and %/100ms. The distribution of low and high WSS and OSI was compared between different spatiotemporal resolutions.

**Results:**

The mean PC-MRI CCA flow (2.5±0.2mL/s) agreed with the ultrasound probe measurements (2.7±0.02mL/s). Mean flow (mL/s) depended only on spatial resolution (CCA:-13%/mm, ICA:-49%/mm). Peak flow (mL/s) depended on both spatial (CCA:-13%/mm, ICA:-17%/mm) and temporal resolution (CCA:-19%/100ms, ICA:-24%/100ms). Mean WSS (Pa) was in inverse relationship only with spatial resolution (CCA:-19%/mm, ICA:-33%/mm). OSI was dependent on spatial resolution for CCA (-26%/mm) and temporal resolution for ICA (-16%/100ms). The regions of low and high WSS and OSI matched for most of the spatiotemporal resolutions (CCA:30/30, ICA:28/30 cases for WSS; CCA:23/30, ICA:29/30 cases for OSI).

**Conclusion:**

We show that both mean flow and mean WSS are independent of temporal resolution. Peak flow and OSI are dependent on both spatial and temporal resolution. However, the magnitude of mean and peak flow, WSS and OSI, and the spatial distribution of OSI and WSS did not exhibit a strong dependency on spatiotemporal resolution.

## Introduction

Atherosclerotic plaques develop at the sites of disturbed flow in the arteries [1, 2]. Besides wall shear stress (WSS) magnitude, some studies show that oscillatory changes of the WSS direction may promote atherogenesis [3–5]. The oscillations within a cardiac cycle are quantified by the oscillatory shear index (OSI) [6, 7]. Although both WSS and OSI contribute to initiation and progression of atherosclerotic disease, most studies focus only on the WSS magnitude and exclude analysis of OSI due to the challenge of obtaining accurate WSS magnitude and OSI simultaneously [8–12].

WSS magnitude is calculated by multiplying blood viscosity with wall shear rate (WSR), the latter being the first radial derivative of blood velocity at the vessel wall. The velocity field in the artery that is necessary to calculate WSR is generally obtained with computational fluid dynamics (CFD). CFD is a powerful simulation tool that enables prediction of blood velocities and related hemodynamic parameters [13, 14]. However, CFD requires accurate boundary conditions, non-clinical expertise and extensive computational resources and time. Alternatively, the velocities can be obtained by phase contrast MRI (PC-MRI) measurements [15–18]. However, the WSS values based on MRI depend on the spatial resolution of PC-MRI [19–23]. In a recent study, we showed that WSS estimates based on in vivo PC-MRI data have a realistic representation of the spatial distribution but underestimate magnitude, due to the limited spatial resolution of PC-MRI [19]. Stalder et al. also investigated the effect of spatial resolution on flow and WSS using synthetic data [17] and showed that the WSS values calculated with the method they proposed were strongly affected by the spatial resolution. Petersson et al. showed that higher true WSS values were underestimated more by PC-MRI and reducing the resolution enhanced the underestimation [20]. These findings suggest that the spatial resolution of PC-MRI measurements should be sufficiently high to obtain the magnitude of WSS accurately. OSI, on the other hand, is a dimensionless parameter which measures the changes of WSS direction over a cardiac cycle. An accurate estimation of OSI might, therefore, only be possible with both sufficiently high spatial and temporal resolutions of PC-MRI measurements.

The MRI settings such as the spatiotemporal resolution involve a trade-off between the measurement duration and the accuracy of the flow, WSS and the OSI estimations. To perform the measurement within clinically feasible scan time, the resolution is generally kept low and the accuracy of these parameters is given away. One can, however, argue that each estimated parameter is affected differently. To our knowledge, none of the previous studies has investigated the effect of spatial and temporal resolution together on these hemodynamic parameters extensively. Our objective was to evaluate the effect of resolution on the assessments of flow, WSS and OSI that we obtained from 2D cine PC-MRI scans of a carotid artery phantom at different spatial and temporal resolutions.

## Methods

### Phantom and flow set-up

**Fig 1:**
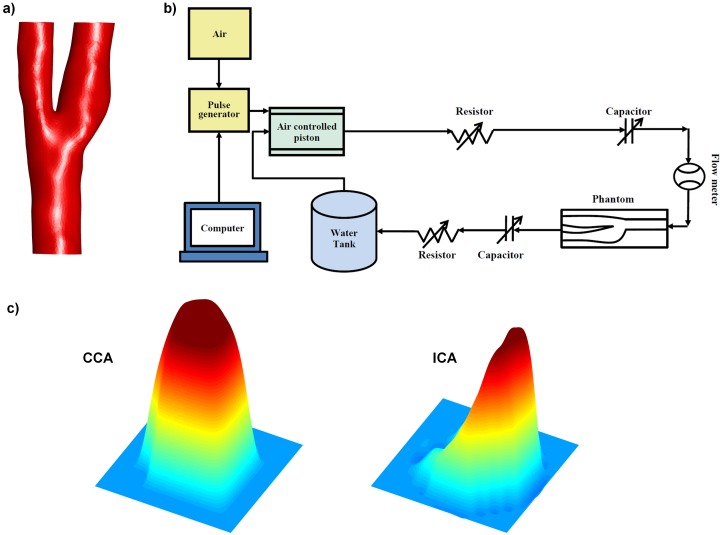
a) The surface reconstruction of the healthy right carotid artery based on which the phantom was built b) The sketch of the pulsatile water flow set-up c) The velocity profile measured at CCA (left) and at ICA (right) measurement planes.

A silicone phantom was built based on the surface reconstruction of a healthy right carotid artery (age 25 years old) acquired from a previous study [19] (Fig 1a). The phantom was connected to a flow set-up (Fig 1b). The set-up consisted of a computer, computer controlled pulse generator, an air pressure controller (LifeTec Group, Eindhoven, The Netherlands) and a closed flow phantom circuit filled with water. The computer, the pulse generator, the air pressure controller, and the flow-meter were placed outside the MRI room. The phantom circuit, including an MR compatible pump system, was placed on the MR table connected to the phantom. The pump system consisted of thin-walled silicone cylinders that were filled with water and embedded in a rigid air-filled enclosure. Air pressure in the rigid enclosure was varied to dilate and contract the water-filled cylinders. One-way valves ensured that this cyclic air pressure induced a pulsatile flow. The shape and the magnitude of the flow waveform were set by adjusting the shape and the amplitude of the cyclic air pressure wave. The shape of the waveform was then tuned by adjustment of resistors and capacitors within the closed fluid circuit. A real-time ultrasound flow probe was used to calibrate the PC-MRI measured flow waveform outside the MRI room before the MRI scans while keeping all experimental conditions the same.

### MRI acquisition

The carotid phantom was scanned with a 3.0T MR system (Ingenia, software version 4.1.3, Philips Healthcare, The Netherlands) using a solenoid rat coil. 2D cine PC-MRI scans were performed at two planes with velocity encoding in 3 directions using various temporal and spatial resolutions as shown in Table 1 (venc: 100 cm/s, TR: 8.9–24.1 ms, TE: 4.67–6.57ms, flip angle: 10°). We performed thirty measurements at different spatial and temporal resolutions at two planes, which took between 1.1 and 21.0 minutes per measurement depending on the spatiotemporal resolution. All measurements were performed on the same day without an interruption of the flow setup to prevent any changes in the pulse shape. The flow stability was verified with the ultrasound flow probe before and after the MR session. One of the planes were chosen in in the common carotid artery (CCA) and the other one in the internal carotid artery (ICA) since the typical velocity profile is closer to a parabolic shape in CCA and it is more skewed in ICA as shown in Fig 1c. To analyze both velocity profiles and to avoid MR table movement in between positions, we chose the first plane 1 cm proximal to the branching point perpendicular to CCA and the second plane 1 cm distal to the branching point perpendicular to ICA. All the acquisitions were performed with retrospective triggering, except those requiring a temporal resolution below 30 ms. For acquisitions with temporal resolution below 30 ms, prospective triggering was used to be able to measure separate flow encoding directions in separate heart cycles. All PC-MRI data was corrected for first-order phase offset errors.

**Table 1:** The spatial and temporal resolution of the PC-MRI measurements.

Measure no.	Spatial resolution [mm]	Temporal resolution [ms]	Measure no.	Spatial resolution [mm]	Temporal resolution [ms]	Measure no.	Spatial resolution [mm]	Temporal resolution [ms]
1	0.2	24.4	11	0.4	100.0	21	0.8	55.6
2	0.3	19.6	12	0.4	142.9	22	0.8	76.9
3	0.3	76.9	13	0.5	11.1	23	0.8	100.0
4	0.3	90.9	14	0.5	38.5	24	0.8	142.9
5	0.3	111.1	15	0.5	58.8	25	1.0	9.1
6	0.3	142.9	16	0.5	76.9	26	1.0	35.7
7	0.4	12.3	17	0.5	100.0	27	1.0	52.6
8	0.4	43.5	18	0.5	142.9	28	1.0	71.4
9	0.4	66.7	19	0.8	9.4	29	1.0	100.0
10	0.4	83.3	20	0.8	35.7	30	1.0	142.9

### Segmentation

The vessels were automatically segmented on the MRI measurement planes by an in-house tool written in MATLAB. Initial segmentation was performed by k-means clustering, followed by an active contour segmentation using the method by Herment [21]. A separate segmentation was performed for each measurement. The segmentation performed on the images at the highest spatial resolution (0.2 mm) will be denoted as the ‘best segmentation’ in the rest of this article.

### Mesh Generation and CFD simulation

The 3D surface of the phantom was reconstructed and the inlet and outlets of the surface were extended in length by 10 diameters. The extended surface was converted into a volume and filled with tetrahedral mesh. The starting size of the mesh elements was 0.2 mm which was gradually increased. The resulting volume mesh included 840725 tetrahedral elements. Time-resolved CFD simulation was performed by using the volume mesh. The echo flow measurement was used as CCA inflow. The velocity profile of CCA was defined as parabolic profile. ECA and ICA-outlets were left as stress free. One flow cycle was 1 sec. and the temporal resolution was defined as 0.01 sec.

### WSS calculations based on PC-MRI

Since the workflow for WSS calculations based on PC-MRI was discussed before in detail, we only give a short overview [22]. Firstly, the inward normal was determined for each point on the surface. The velocities measured by PC-MRI were interpolated along the inward normal direction at 2 points at a distance of 1.5 and 3 mm from the wall [22]. The velocity at the surface was set to zero, and a spline curve was fitted through these velocity vectors including the zero velocity point at the wall. By taking the gradient of the curve at the point on the wall, wall shear rate (WSR) was calculated. WSS was calculated by multiplying WSR with the dynamic viscosity of water which was assumed to be 1.0·10^−3^ Pa·s.

### OSI Calculations

The commonly used definition of OSI was introduced by He et al. [7] as follows:

$$OSI\left(\vec{s}\right)=0.5\left[1-\frac{\left|\sum_{0}^{T}WSS\left(\vec{s},t\right)\Delta t\right|}{\sum_{0}^{T}\left|WSS(\vec{s},t)\right|\Delta t}\right]$$ (1)

where $\vec{s}$ is the position at the vessel wall, t is the time point, Δ*t* is time step, and T is the number of time steps within one cardiac cycle. The OSI varies between 0 and 0.5 where higher OSI indicates larger changes in the WSS direction.

### Analysis

The flow waveforms measured in the CCA were compared with the ultrasound flow probe measurements. For different spatiotemporal resolutions, we analyzed cross-sectional area, mean flow, peak flow, WSS, and OSI at the CCA and ICA. The WSS values were firstly averaged over the cardiac cycle and subsequently over the circumference of the vessel wall. The OSI values were averaged over the circumference of the vessel wall. Furthermore, WSS and OSI values were averaged separately over the quarters of the vessels to study the local distribution of WSS and OSI over the circumference. The WSS and OSI values were also obtained by CFD at the same-cross-sections of the CCA and ICA and compared with those obtained by PC-MRI measurements. Finally, to investigate the effect of segmentation on the estimated hemodynamic parameters, the ‘best segmentation’ was applied to each dataset. The mean flow, WSS and OSI were obtained with the best segmentation and compared with those obtained with the segmentation per measurement.

### Statistical analysis

The associations between spatiotemporal resolutions and the hemodynamic parameters and between the results based on the measurement-specific segmentations and best segmentation were tested by linear regression analysis. In statistical evaluations, the level of significance was chosen at p<0.05. The results were presented as the mean ± standard deviation of the 30 measurements.

## Results

The linear regression coefficients and the slopes of the linear regression lines (in %/mm and %/100ms) between the hemodynamic parameters and spatiotemporal resolution are summarized in Table 2.

**Table 2:** The regression analysis for different spatiotemporal resolutions and the hemodynamic parameters.

		Spatial resolution		Temporal resolution	
		r^2^	Slope (%/mm)	r^2^	Slope (%/100ms)
CCA	Mean flow [mL/s]	0.32	-13.0%*	0.00	-0.4%^NS^
	Peak flow [mL/s]	0.13	-13.0%^NS^	0.66	-19.0%*
	WSS [Pa]	0.36	-19.0%*	0.09	-6.0% ^NS^
	OSI	0.16	-26.0%*	0.10	-13.0% ^NS^
ICA	Mean flow [mL/s]	0.70	-49.0%*	0.00	-1.4% ^NS^
	Peak flow [mL/s]	0.15	-17.0%*	0.44	-24.0%*
	WSS [Pa]	0.64	-33.0%*	0.04	-5.6% ^NS^
	OSI	0.06	-16.0%^NS^	0.16	-16.0%*

The slopes (in %/mm and %/100ms) were calculated by dividing flow, WSS and OSI with their respective maxima.

(*) indicates that p<0.05,

(^NS^) indicates p> = 0.05.

### Flow waveform at CCA

**Fig 2:**
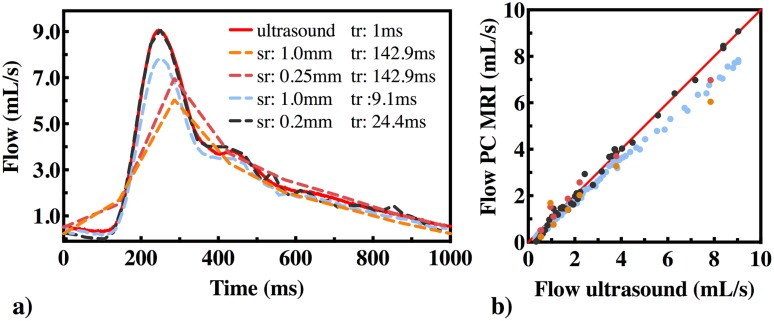
a) Flow waveforms at different spatial(sr) and temporal(tr) resolutions. Dashed lines show the PC-MRI measurements and the red line shows the ultrasound probe measurement. Spatial resolution varied between 0.2mm and 1 mm and the temporal resolution varied between 9.1 ms and 142.9 ms. b) Ultrasound probe vs. PC-MRI flow measurements.

Flow waveforms obtained from PC-MRI measurements at the highest and the lowest spatial and temporal resolutions are shown in Fig 2a. The red line shows the flow waveform based on the ultrasound probe measurement. At the highest spatial resolution (0.2 mm) and the highest temporal resolution (24.4 ms, black dashed line), the shape of the flow waveform was similar to the one measured by the ultrasound probe. At the lowest spatial resolution (1.0 mm) and the highest temporal resolution (9.1 ms, light blue dashed line), the shape of the flow waveform was still captured although peak flow was underestimated (7.8 mL/s). At lowest temporal resolution (142.9 ms, red and orange dashed lines), the peak flow was shifted backward in the cardiac cycle and underestimated. The ultrasound probe measurements were plotted against the PC-MRI measured flows in Fig 2b which shows underestimation of flow at higher flows in all cases except for the measurement at the highest spatial and temporal resolution.

### Area, mean flow, and peak flow at CCA and ICA

**Fig 3:**
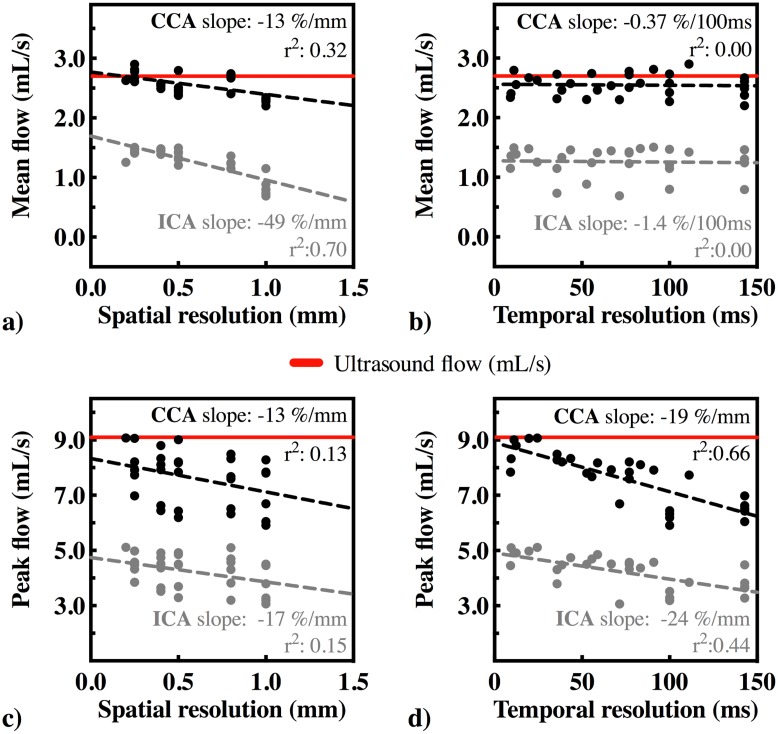
a) Mean flow [mL/s] vs. spatial resolution [mm] b) Mean flow [mL/s] vs. temporal resolution [ms] c) Peak flow [mL/s] vs. spatial resolution and d) Peak flow [mL/s] vs. temporal resolution at the CCA and ICA. Red lines show the mean and peak flow measured by ultrasound probe.

The cross-sectional area was 24.6 ± 0.6 mm^2^ at the CCA and 29.0 ± 2.9 mm^2^ at the ICA. The mean flow based on PC-MRI measurements was 2.5 ± 0.2 mL/s at the CCA and 1.3 ± 0.2 mL/s at the ICA. The mean flow vs. the different spatial resolutions is plotted in Fig 3a and the mean flow vs. the different temporal resolutions in Fig 3b. A significant association was found between mean flow and the spatial resolution (slope -13.0%/mm for the CCA and -49.0%/mm for the ICA). No correlation was observed between mean flow and temporal resolution. The mean flow based on the ultrasound flow probe measurement was 2.7 ± 0.02 mL/s at the CCA; hence, the ratio of the mean flow based on PC-MRI measurements and the ultrasound flow probe measurement was 95.1 ± 7.9%.

The peak flow was 7.6 ± 1.0mL/s at the CCA and 4.2 ± 0.6 mL/s at the ICA. The peak flow based on ultrasound probe flow measurement was 9.1 mL/s. At the highest spatial resolution (0.2 mm) and the highest temporal resolution (24.4 ms), the peak flow was estimated accurately (9.1 mL/s) At lower spatiotemporal resolutions, peak flow was underestimated. The estimated peak flow was significantly dependent on both spatial (-17.0%/100ms for ICA) and temporal resolution (-19.0%/100ms for the CCA to -24.0%/100ms for the ICA). The peak flow vs. spatial resolution is shown in Fig 3c and the peak flow vs. temporal resolution is shown in Fig 3d.

### WSS at CCA and ICA

**Fig 4:**
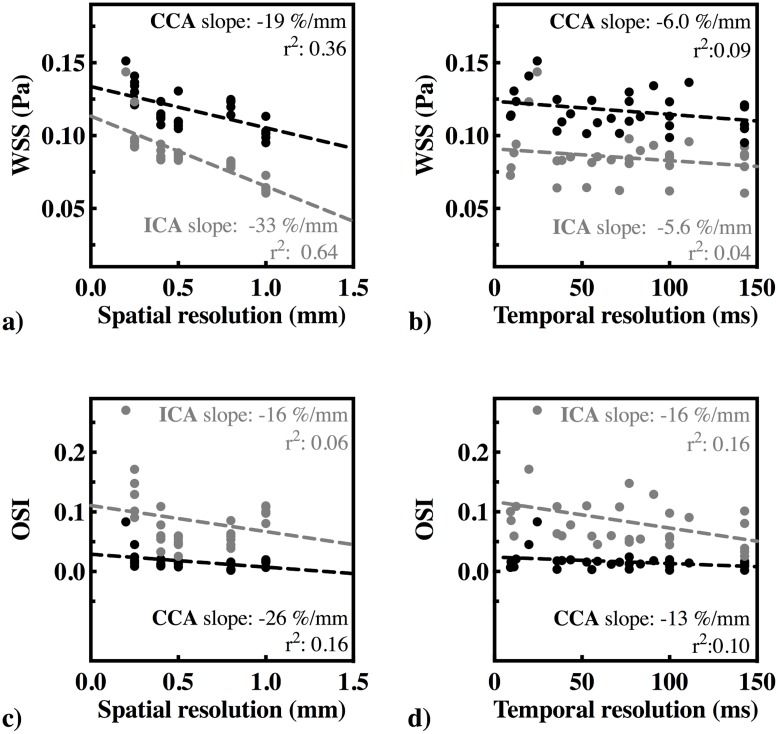
a) WSS [Pa] vs. spatial resolution [mm] b) WSS [Pa] vs. temporal resolution [ms] c) OSI vs. spatial resolution and d) OSI vs. temporal resolution.

The WSS at different spatial and temporal resolutions are shown in Fig 4a and 4b. The WSS was 0.12 ± 0.01 Pa at the CCA and 0.09 ± 0.02 Pa at the ICA based on the PC-MRI measurements. WSS based on CFD was 0.14 Pa at the CCA and 0.17 Pa at the ICA. At the highest spatial resolution (0.2 mm) and the highest temporal resolution (24.4 ms), WSS was 0.15 Pa at the CCA and 0.14 Pa at the ICA. At lower spatial resolutions, the estimated WSS was lower. We found a significant inverse relationship between the estimated WSS and the spatial resolution (-19.0%/mm for the CCA and -33.0%/mm for the ICA). No relationship was found between mean WSS and temporal resolution.

### OSI at CCA and ICA

Fig 4c and 4d show OSI at different spatial and temporal resolutions for the CCA and ICA. OSI was found 0.02 ± 0.02 at the CCA and 0.08 ± 0.05 at the ICA based on the PC-MRI measurements. The OSI based on CFD was 0.03 at the CCA and 0.07 at the ICA. The highest OSI values were found at the highest spatial resolution (0.2 mm) and highest temporal resolution (24.4 ms), which were 0.08 for the CCA and 0.27 for the ICA. The estimated OSI was lower at lower spatiotemporal resolutions. We found a significant association between OSI and spatial resolution in the CCA (-26.0%/mm), but the association between OSI and temporal resolution was not significant in the CCA. In the ICA, we only found a significant association between OSI and temporal resolution (-16.0%/100ms).

### Local WSS distribution

**Fig 5:**
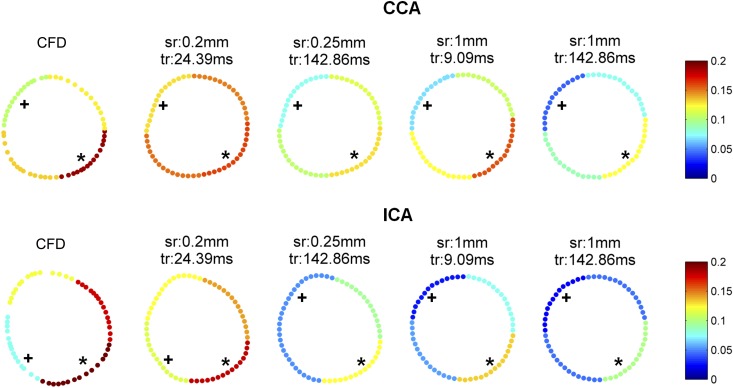
The mean WSS [Pa] of each quarter by CFD and by PC-MRI measurements at different spatial and temporal resolutions in the CCA and ICA. * shows the highest WSS quarter and + shows the lowest WSS quarter.

The mean WSS of each quarter for four measurements in the CCA and the ICA is shown in Fig 5. For the CCA, the highest WSS quarter was the bottom right quarter (0.14 ± 0.01 Pa) and the lowest WSS quarter was the top left quarter (0.07 ± 0.02 Pa). These highest and the lowest WSS regions of the CCA were found in all measurements regardless of spatial and temporal resolution. Based on CFD calculations, the bottom right quarter was also found as the highest WSS quarter (0.19 Pa) and the top left quarter was the lowest WSS quarter (0.11 Pa) for the CCA. For the ICA, the highest WSS quarter was the bottom right quarter which was found in all measurements (0.13 ± 0.02 Pa) and also in CFD calculation (0.30 Pa). The lowest WSS quarter for the ICA was the top left quarter (0.04 ± 0.02 Pa) in 28/30 measurements (93%). The lowest WSS quarter in the ICA was the bottom left quarter (0.08 Pa) based on CFD calculations and the WSS at the top left quarter was 0.12 Pa.

### Local OSI distribution

**Fig 6:**
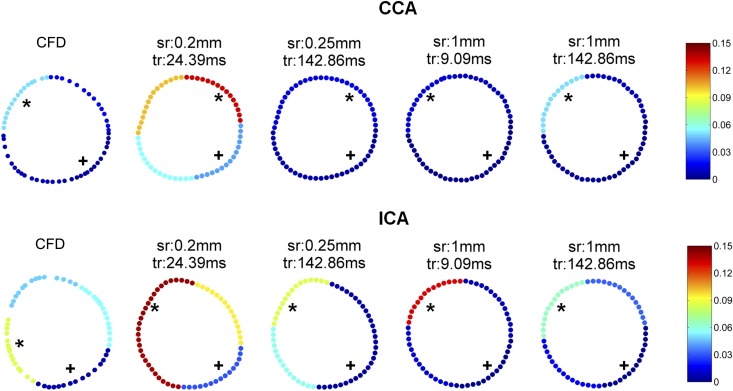
The mean OSI of each quarter by CFD and by PC-MRI measurements at 4 different spatial and temporal resolutions in the CCA and ICA. * shows the highest OSI quarter and + shows the lowest OSI quarter.

The mean OSI of each quarter for four measurements is shown in Fig 6. OSI was generally low in all quarters of the CCA. The highest OSI quarter in the CCA was the top left quarter (0.04 ± 0.02) which was found in 23/30 measurements (77%) and in CFD calculation (0.07). The lowest OSI quarter was the bottom right quarter in 29/30 (97%) measurements (0 ± 0.01) and in also CFD calculation (0.0). For the ICA, the lowest OSI quarter was the bottom right quarter which was found in all measurements (0.01 ± 0.01) and also in CFD calculation (0.01). The highest OSI quarter was the top left quarter (0.20 ± 0.08) in all measurements regardless of spatial and temporal resolution. CFD based OSI was lower in the ICA. The highest OSI quarter (0.12) was the bottom left quarter and the OSI at the top left quarter was 0.07.

### Calculations using fixed segmentation at CCA

The best segmentation resulted in the cross-sectional area of 24.7 mm^2^ for the CCA. The results based on the best segmentation were very similar to those based on the segmentations per measurement. The flow obtained with the best segmentation was 2.7 ± 0.2 mL/s which was in agreement with the ultrasound probe flow measurements and 8.0% higher than that obtained with the measurement-specific segmentations (2.5± 0.2 mL/s, r^2^ = 0.85, p<0.001). We found no significant correlation between mean flow and spatial resolution after switching to the best segmentation (r = -0.09, p = 0.62). The WSS with the best segmentation showed good agreement with that based on the measurement-specific segmentations (r^2^ = 0.99), which were on average only 2% lower in magnitude (0.11 ± 0.01 Pa, p<0.001). The mean OSI based on the best segmentation was 0.02 ± 0.02 which was also in good agreement with that based on the measurement specific segmentations (r^2^ = 0.99).

## Discussion

In this study, we investigated the influence of spatial and temporal resolution on the estimation of mean flow, peak flow, WSS and OSI in a realistic phantom of a carotid bifurcation. Our results show that not all parameters are affected to the same extent by spatial and temporal resolution. For example, mean flow was not dependent on temporal resolution; but it was influenced by spatial resolution. This was caused mainly by the difference in segmentation. At lower spatial resolutions, delineating the borders of the cross-sectional area was more difficult with larger voxels around the vessel wall. In fact, applying the best segmentation improved the estimation of mean flow for all resolutions. Nevertheless, mean flow was estimated accurately with less than 10% error regardless of segmentation, spatial and temporal resolution. These observations correspond well with previous literature that also showed correct flow rates for lower resolution scans [17]. Please note that correct flow quantification requires a minimum number of 3–4.5 voxels/diameter [24, 25].

The estimated flow waveform and the peak flow were mainly dependent on temporal resolution. To obtain the peak systolic time point within the cardiac cycle and the peak systolic flow accurately, it was necessary to perform the acquisition at a high temporal resolution. The higher temporal resolution also reduced flattening of the flow waveform. At a temporal resolution lower than 50 ms, the error in the estimated peak flow was still more than 10%.

The WSS values were specifically relying on high spatial resolution. The observed effects of spatial resolution on average WSS magnitude are in correspondence with existing literature. The typical underestimation of WSS, when quantified by PC-MRI, has been described extensively [17, 20, 22, 26, 27]. In the CCA, the mean WSS based on CFD was found similar to that based on the PC-MRI measurement at the highest spatiotemporal resolution. At lower spatial resolutions, WSS was underestimated. The mean WSS in the ICA was underestimated by all PC-MRI measurements possibly caused by the skewed velocity profile. To calculate PC-MRI based WSS, we used velocities interpolated at the fixed distances to the wall points. Hence, the high slope of the skewed velocity profile might have been omitted, resulting in larger underestimation at higher WSS region. At lower spatial resolutions, the changes in the spatial resolution had only a marginal impact in the estimated WSS value, e.g. a decrease of 0.1 mm in spatial resolution only decreases the WSS by 2–3%. Note that the duration of our 2D PC-MRI measurements at spatial resolution of 0.2 mm and temporal resolution of 24 ms was 21 minutes at only one plane which is not feasible in the clinic. Furthermore, the noise level increases with the increase of spatial resolution, even more if a standard receive coil is used. Nevertheless, recent developments in MRI acceleration technologies will lead to shorter scan times and/or decreased noise levels at high resolutions [28, 29], which, in time, will allow faster and more accurate WSS based on PC-MRI.

The effects of temporal resolution on time-resolved WSS parameters have not been investigated previously. We found that the WSS values averaged over the cardiac cycle were not dependent on the temporal resolution. For the CCA, OSI was dependent on spatial resolution, but not on temporal resolution. The CFD based OSI was in good agreement with PC-MRI measurements in the CCA. For the ICA, OSI was dependent on temporal resolution, but not on spatial resolution. This is likely due to the low OSI in the CCA and the high OSI in the ICA. OSI values at high spatial and temporal resolutions were higher than those based on CFD which might have been caused by the increased level of noise relative to the signal. However, at lower spatiotemporal resolutions, the changes in the spatiotemporal resolutions affect the estimated OSI values only to a limited extent.

Despite underestimation of the WSS and OSI magnitude, the low and high WSS and OSI regions showed a good agreement in most of the measurements, regardless of spatiotemporal resolution. This result together with the limited dependency of WSS and OSI values on the chosen spatiotemporal resolution indicates that WSS and OSI can be compared between studies with similar PC-MRI protocols.

Although the segmentation had an influence on the estimated flow, the effect of segmentation on WSS and OSI was found to be small. This may be related to the fact that choosing zero velocity at the wall improves the robustness of WSS estimations, as shown by Petersson et al [20].

This study had three main limitations. Firstly, we limited the study to only 2D PC-MRI measurements (with 3D velocity encoding) within the carotid artery. We chose to perform 2D acquisition to keep the MRI scans within clinically acceptable scan times since high-resolution 4D PC-MRI measurements would result in unacceptable long scan times. To overcome this limitation, we chose two MRI measurement planes, one at CCA, and one at ICA. The chosen measurement planes were close to the carotid bifurcation and they were at the regions prone to plaque development. Hence, these cross-sections are of clinical interest. In addition, the velocity profiles at the CCA and ICA planes were the two typical velocity profiles present in carotid artery flow. Therefore, the effect of spatiotemporal resolution that we observed in these two cross-sections is applicable to WSS and OSI in carotid arteries and in other vessels with similar velocity profiles. Secondly, we performed only in vitro measurements, which do not necessarily represent in vivo situation. However, the long scan times would again be the limitation to perform measurements at very high spatial and temporal resolutions. Finally, we used water as the medium instead of a blood representing fluid. This resulted in lower WSS values than the physiological WSS values. However, wall shear rate we measured is expected to be in the range of physiological values and the effect of blood viscosity on the estimated WSS values was beyond the scope of this study.

## Conclusions

In this study, we showed that the hemodynamic parameters such as mean flow, peak flow, flow waveform, WSS and OSI are influenced by spatial and temporal resolution of PC-MRI measurements but to different extents. The mean flow is dependent on the spatial resolution which is caused by the segmentation errors. However, the effect of spatial resolution on the mean flow is small. We show that both mean flow and mean WSS are independent of temporal resolution. WSS is more sensitive to spatial resolution, while OSI is sensitive to both spatial and temporal resolution. Nevertheless, this study shows that the magnitude of mean and peak flow, WSS and OSI as well as the location of low and high WSS did not exhibit a strong dependency on the spatiotemporal resolution of the measurement.

## Supporting Information

S1 Fig Mean flow at different spatial and temporal resolutions. Red circles show the measurement points. White lines show the PC-MRI measurement durations of 18, 6 and 2 minutes (left to right). Top: CCA and bottom: ICA.
(TIF)

S2 Fig Peak flow at different spatial and temporal resolutions. Red circles show the measurement points. White lines show the PC-MRI measurement durations of 18, 6 and 2 minutes (left to right). Top: CCA and bottom: ICA.
(TIF)

S3 Fig WSS at different spatial and temporal resolutions. Red circles show the measurement points. White lines show the PC-MRI measurement durations of 18, 6 and 2 minutes (left to right). Top: CCA and bottom: ICA.
(TIF)

S4 Fig OSI at different spatial and temporal resolutions. Red circles show the measurement points. White lines show the PC-MRI measurement durations of 18, 6 and 2 minutes (left to right). Top: CCA and bottom: ICA.
(TIF)

S1 File The raw material and the analysis files. This file includes echo flow measurements, analysis of mean and peak flow, WSS and OSI the calculations based on best segmentation and varying segmentation.
(ZIP)
