# Supplementary figures and images for: The Effect of Spatial and Temporal Resolution of Cine Phase Contrast MRI on Wall Shear Stress and Oscillatory Shear Index Assessment
Source: PLoS One. 2016 Sep 26;11(9):e0163316. doi: 10.1371/journal.pone.0163316 (PMC5036833; doi:10.1371/journal.pone.0163316)

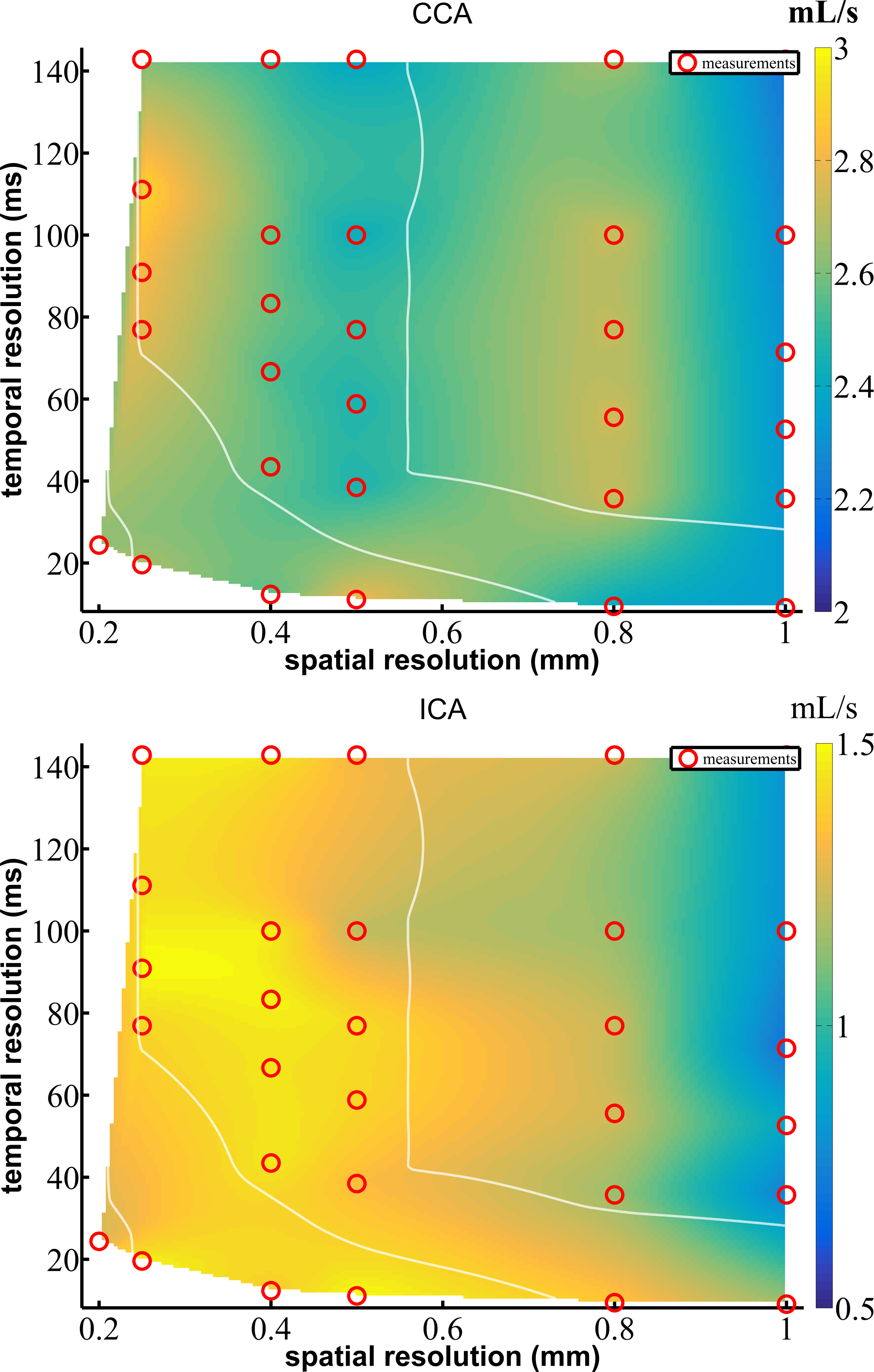

Supplement: S1 Fig — Red circles show the measurement points. White lines show the PC-MRI measurement durations of 18, 6 and 2 minutes (left to right). Top: CCA and bottom: ICA. (TIF) [file pone.0163316.s001.tif]

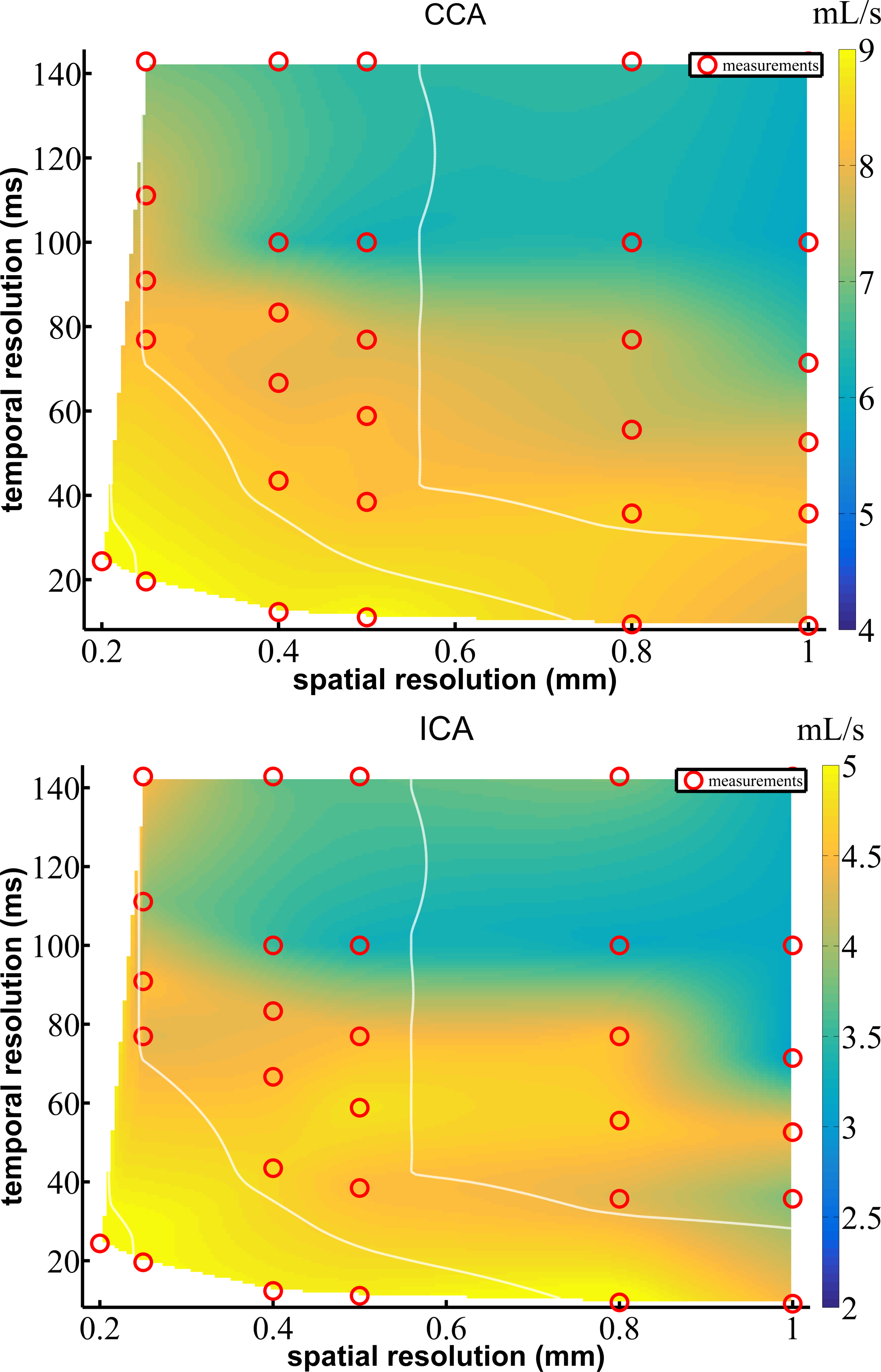

Supplement: S2 Fig — Red circles show the measurement points. White lines show the PC-MRI measurement durations of 18, 6 and 2 minutes (left to right). Top: CCA and bottom: ICA. (TIF) [file pone.0163316.s002.tif]

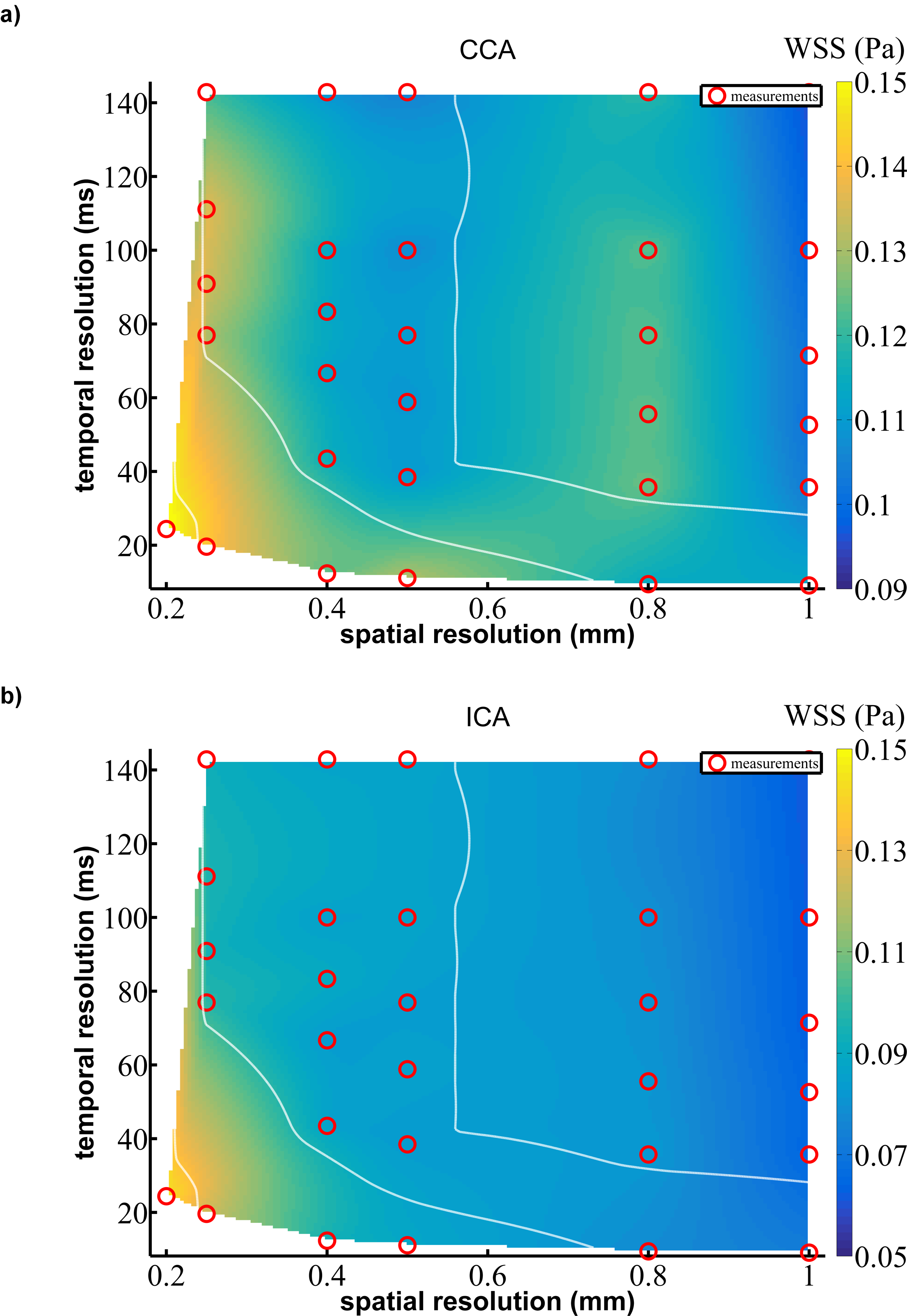

Supplement: S3 Fig — Red circles show the measurement points. White lines show the PC-MRI measurement durations of 18, 6 and 2 minutes (left to right). Top: CCA and bottom: ICA. (TIF) [file pone.0163316.s003.tif]

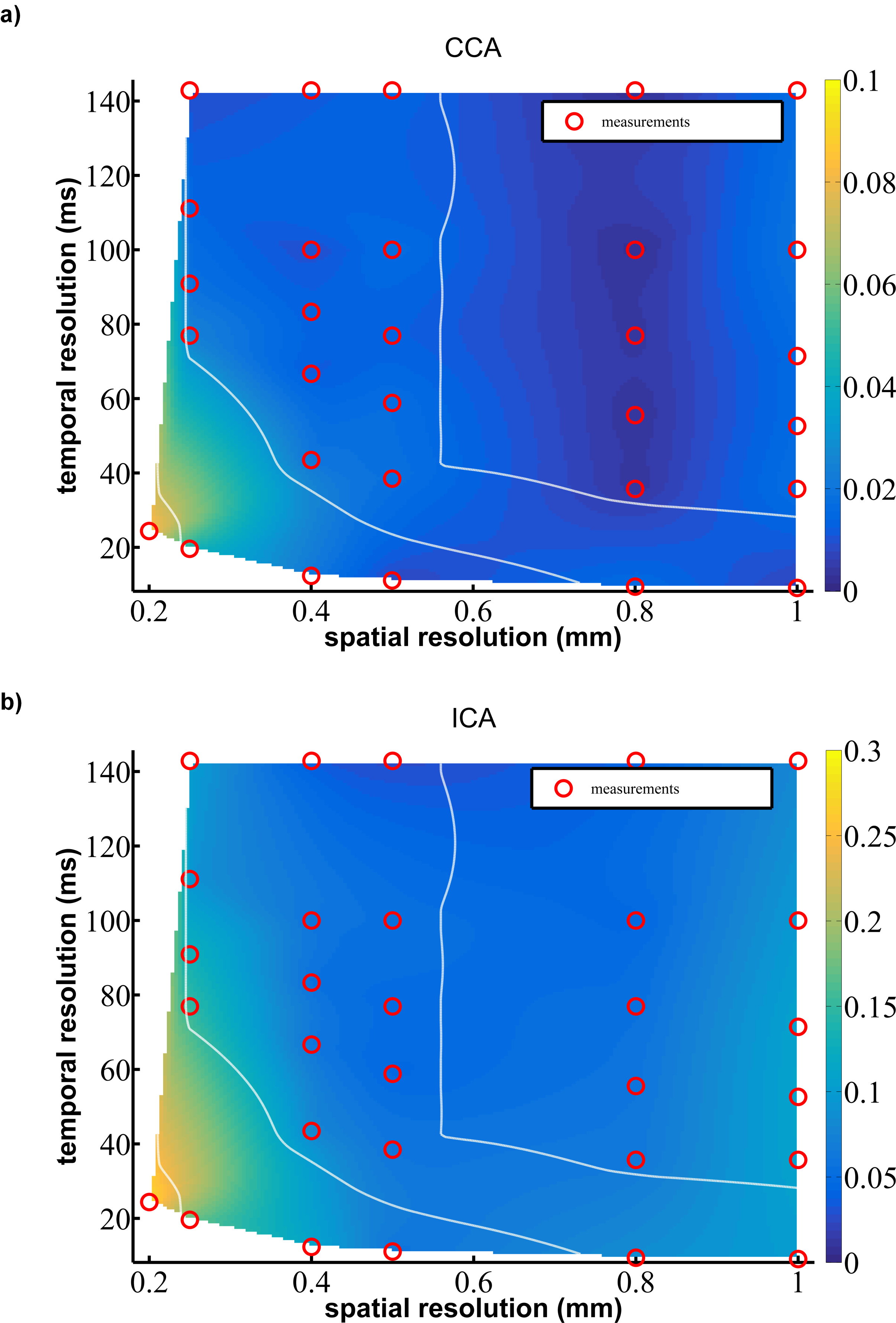

Supplement: S4 Fig — Red circles show the measurement points. White lines show the PC-MRI measurement durations of 18, 6 and 2 minutes (left to right). Top: CCA and bottom: ICA. (TIF) [file pone.0163316.s004.tif]
